# Slaughter-house Inspection1Presented before the United States Veterinary Medical Association, Omaha, Nebraska, September, 1898.

**Published:** 1898-11

**Authors:** S. Stewart

**Affiliations:** Kansas City, Kan.


### SLAUGHTER-HOUSE INSPECTION.[1]

By S. Stewart, M.D., D.V.M.,
KANSAS CITY, KAN.

The establishment of slaughter-house inspection by the government at the large slaughtering-establishments doing a foreign and interstate business has served to attract attention to the necessity for careful inspection at all slaughter-houses supplying cities and towns with animal food-products.

In order that slaughter-house inspection may be all that public health requires that it should be several conditions must be harmoniously conjoined: There must be legal authority to conduct inspection and enable the inspector to enforce necessary regulations; there must be a substantial moral support on the part of the public, both general and official; the inspector must be thoroughly competent.

If inspection be established under the authority of a local or State board of health, the power for enforcement of its rules is

[1] Presented before the United States Veterinary Medical Association, Omaha, Nebraska, September, 1898.

usually ample and easily applied. When the authority for inspection is through municipal ordinance political influence is a forceful factor, often rendering uncertain the official position of the inspector, and not infrequently determining a vacillating and biased service, with laxness where financial or political force is applied.

If inspection be intelligently and honestly conducted, and the public kept informed as to the work done through official reports, public sentiment will lend strong moral support to this kind of sanitary service. No one thing will create a stronger public approval and more general satisfaction than the assurance that the food upon the table is not tainted with disease.

The qualifications of the inspector for the duties of his office are important factors in the establishment and maintenance of a service which fully protects the public and yet does justice to the owners of animals slaughtered. He should possess a thorough knowledge of the anatomy of domestic animals and have a good working knowledge of comparative pathology. He should be familiar with the antemortem symptoms and postmortem lesions of the more common diseases, and possess the mental acumen to trace out and determine the rarer ones, and withal to judge early the influence any disease or morbid condition may have on the wholesomeness, as human food, of the flesh of an affected animal. He must be honest, courteous, and discreet. Inspection may be carried on without serious conflict with the slaughterer if the inspector condemns with discretion, and has the tact to explain, in simple language, why he condemns when objection is raised by the butcher or owner. In this way he may be led to have confidence in the inspector, and will manfully bear his losses when unfit carcasses or organs are condemned as unfit for food and destroyed.

The carcasses of animals affected with anthrax, rabies, and septic conditions may be classed as positively dangerous, both for food and to handle; those affected with tuberculosis, actinomycosis, Texas fever, erysipelas, sheep-pox, hog-cholera, and swine-plague, or any disease producing elevation of temperature, as dangerous and suspiciously unwholesome; also beasts which have died before slaughter, or must be killed to save them, and flesh saturated with œdematous fluid and blood.

There is a class of meats which is decidedly disgusting and loathsome, though not positively harmful as food, such as the flesh of animals which were drowned, smothered, or died of apoplexy; females in the parturient state, or near its approach; unborn or recently-born young; animals fed on loathsome offal; flesh which emits an unpleasant odor; and flesh containing parasites, such as trichinæ and cysticerci, the last two being harmful if consumed raw.

Flesh may be considered wholesome in cases of recent injury, localized diseases of single organs, of a chronic, non-malignant character, or localized parasitic invasion, the parts involved having been removed.

The interest of the owner of meats under inspection is to be considered in connection with the health and prejudice of the consuming public. You will observe that popular and personal prejudices play quite an important rôle in this connection. Persons not accustomed to seeing animals slaughtered and the parts or organs prepared for food are often disgusted with many conditions and products which are perfectly wholesome; and others, who do this work, or constantly see it done, become accustomed to and consider wholesome many conditions of flesh which may be decidedly harmful or loathsome to the general public. The public should be properly protected from the ignorance or rapacity of the butcher, and the owner and slaughterer of animals be protected from the ignorant prejudices of the public.

Examinations made before slaughter are highly important. Considerable enlargements of any of the tissues about the head, neck, and limbs are easily discernible; gangrenous wounds and skin-diseases will be noticed, and the sick or bruised animal which gets out to one side by itself, or lies down, while the others stand or walk about, will not be overlooked; also the class known as "downers," or cripples, which cannot or will not walk to the slaughter-house, may be seen. Special attention should be given to such animals during the postmortem inspection, for the wily butcher knows how to cut away evidences of disease skilfully while removing the skin, limbs, and head.

If the slaughtering-establishment be a small one, and the examiner has abundant time to watch the entire process of slaughter leisurely, no abnormal or diseased condition need escape his notice; but in large abattoirs, where 100, 200, or even 500 animals are slaughtered per hour, the process of dressing is done in parts, at several points along the journey of the carcass from the killing-bed to the refrigerator. The head, feet, and visceral organs are quickly removed to another part of the establishment, so the evidence of disease must be seen quickly, if at all, and the carcass of which they were a part identified. Still greater acumen must be exercised in small abattoirs, where the carcass is dressed and the viscera set aside to be examined at the convenience of the inspector.

During the transportation of cattle by railroad to the markets many are injured. These injuries vary from a slight bruise to extensive contusions of the soft parts and fracture of the ribs, vertebræ, or bones of the extremities. Ten to thirty hours after infliction these injuries are manifest in the live animal by swelling and puffiness over the seat of contusions, and, if extensive, the animal moves about very stiffly, as though foundered. If the ribs are broken the injured side is protected as much as possible by muscular rigidity on that side. If a femur, ilium, or vertebra is fractured, the animal will be unable to rise, and must be hauled from the car or yards to the slaughter-house in a cart. Sometimes cattle get down in an overloaded car and cannot get up, owing to the crowded condition of the car. They are trampled on by the others and, after much struggling, become discouraged and will not get up; or, during a rainy or an icy period, cattle slip, violently separating the hind legs at right angles to the median line of the body, rupturing the muscles in the pubic region, perhaps fracturing or dislocating the femur or other bones. These animals are known as "downers."

When slaughtered, the cases of severe injury of more than twenty-four hours' standing do not bleed so freely nor so perfectly as a sound animal. When the skin is removed contusions recently inflicted are easily discerned, the subcutaneous connective tissue and fat being infiltrated with blood escaped from ruptured capillary vessels. If the contusions be extensive, as when a bullock gets down in a car and is repeatedly trampled upon, the fat and connective tissue of the back and sides of the body are torn and pulpified to such a degree that the skin is removed from the injured parts by very slight traction, and the surface of the body is discolored over large areas. The contusions may extend deeply into the muscular structures, even through the thoracic or abdominal walls, being accompanied by hemorrhage into the parts, pulpification of the muscle structures, and sometimes fracture of ribs.

If the injured animal be not slaughtered before febrile conditions are established, the injured tissues become infiltrated with an exudate, varying in color and consistence from a gelatinous amber-colored serum to a thin, dirty fluid, with sometimes a disagreeable odor. The expert butcher deftly removes the hemorrhage, stained, infiltrated, and torn fat and connective tissue overlying the muscular structures, also the superficial muscles, if coagulated blood and serum be found in and beneath them; then with a brush and plenty of very hot water the smaller hemorrhagic discolorizations are softened and washed away, the parts presenting a nearly normal color when dried and placed in a refrigerator until thoroughly cooled.

When the injuries are recent, as indicated by the absence of the products of inflammation, the bruised and torn parts can be cut away, leaving the remainder of the carcass wholesome food and not offensive in appearance. In those cases where the serous exudate is extensive or malodorous, or where rigor mortis is established before the skin can be removed, changes have taken place in the fluid and solid tissues which render them suspiciously—if not certainly—dangerous for food, and they should be condemned.

The class mentioned as "downers" or cripples naturally require especial attention, yet when slaughtered and dressed it is often difficult to discern any sufficient reason why some would not or could not walk to the shambles, so slight are the tangible lesions. Many of them present lesions of the bones and contiguous soft parts, and the same principles apply in determining the wholesomeness of the flesh as though these animals walked to the slaughter-house.

Large suppurating wounds from punctures, gunshots, or branding-irons, or gangrenous wounds are sufficient cause for rejecting the bearers for food purposes. Such animals are usually in a declining physical condition, which fact makes it highly suspicious, if not certain, that the structures of the body are deleteriously influenced by poisonous elements carried from such wounds. If such wounds be small, and postmortem sections of the surrounding structures and contiguous glands show them to be normal in color and consistence, the removal of a liberal portion of the structure surrounding such wound should render the carcass unobjectionable.

Actinomycosis is the most prevalent disease of cattle in this section of the country, and in at least 80 per cent. of the cases in cattle offered for sale at the market the lesions of the malady are confined to the structures of the head and neck. Indeed, in most cases it is a local affection, in no way affecting the general system, excepting as it interferes with prehension, mastication, and deglutition of food. The majority of cases involve the inferior or superior maxillary bones. In all these cases coming under my notice one or more fistulous tracts were found discharging into the cavity of the mouth. These fistulæ are to be found before the disease has reached sufficient development to be observable by visual examination externally, and long before the overlying skin has become involved in any degree. A careful examination of the internal organs of many cattle having actinomycosis of the maxilla in the early stage, but with fistulæ opening into the mouth, has failed to reveal the involvement of any visceral organ. As stated before, the lesions of this disease are usually in the tissues about the head and neck. When the soft structures only are involved it usually begins in one or more of a chain of lymphatic nodes, extending from the mouth to the thorax, most frequently in the submaxillary region. The disease processes set up in these nodes destroy them, and in their stead is developed a dense, thick-walled sac of variable size, containing a whitish, odorless fluid or semifluid mass, which, it is claimed, "consists of detritis resembling pus, but lacking the specific micrococci which are always present in pus." The skin over these tumors may be involved and a fistula established, the external end of which is surrounded by a granular growth, which necroses on the surface, giving off a very offensive odor and having a disgusting appearance.

Extension to other parts is more frequent when the disease is glandular in character. In about 20 per cent. of cases the fungus invades the soft parts about the head and neck; many of these present actinomycotic growths in the lungs, and occasionally the liver and intestinal structures are invaded. The disease appears to extend along the lymphatic channels rather than to find dissemination through the general circulation. Out of several thousands of cases only two were reported as generalized, and careful inquiry showed no involvement of the muscular structures, but was confined to the head, neck, and glandular structures of the cavities; and I question if these cases were not pyæmia coincident with actinomycosis. Actinomycosis of the tongue is very rare.

When the bony structures are the parts invaded by the actinomyces, the characteristic proliferation of the osseous and perosseous tissues attain dimensions which give it the popular name of "big jaw." The fungus destroys the bone and its covering, supplanting them with a new growth of fibrous tissue, enclosing masses of granular tissue, in which are embedded small yellowish points of gritty, purulent fluid, the hard grains being clumps of the specific microörganism of actinomycosis. When the overlying skin is involved the surface presents one or more granuloma which surrounds fistulous openings. If these growths have not interfered with the general health of the animal, sentiment is the most tangible reason for condemning the carcass.

Tuberculosis is found in a small percentage of the cattle slaughtered in the valley of the Missouri, being found principally in cows over five years old, but is occasionally found in calves and young cattle. No structure of the body is entirely exempt from disease-processes set up by the tubercle bacilli, but there is a much greater tendency for these germs to establish themselves in the lungs, thoracic glands, and mesenteric glands, and then spread to other contiguous organs and tissues, or becoming generally disseminated throughout the body. These bacilli, by their active presence in a tissue, induce a new growth about them, which, if it be near or on the surface, projects or stands out like granules. These growths are called "tubercles." They are found scattered through the substance of glands and other structures, or on their surfaces. This form of development in the serous membranes constitutes what is known as the "pearl" disease. Tubercles are often agglomerated into masses from the size of a pea to an egg, and even attain the weight of ten pounds or more. Tubercles or masses when found on free surfaces have the appearance of granulation tissue, but when cut across the centres are found to consist of semi-solid, whitish, caseous material, and in chronic cases may contain small particles of lime-salts, giving this cheese-like substance a gritty feeling to the touch. Sometimes the necrosed tissue in the tuberculous glands of the neck and thorax and in large tubercular masses in the substance of the lungs and liver may be liquid or partly liquid.

It is a very difficult matter to detect the tuberculous animal when confined with others in slaughter-house yards, as there are no pathognomonic signs which plainly and certainly distinguish it from the non-tuberculous, unless it be in the cow bearing the tuberculous udder and contiguous lymphatic glands. By tactile examination any considerable development of tubercles in the lymphatic glands above and behind the udder, or in the udder itself, may be recognized by the nodular character of the induration present, the non-tubercular induration of this organ giving a more uniformly smooth surface to the touch.

Upon postmortem examination the observer will readily discover, when present, the granular appearing tubercular growths on the serous surface covering any viscus or lining the thoracic or peritoneal cavity. I know of no normal or pathological condition presenting a similar appearance. These growths are nearly always present either in the thoracic or peritoneal cavity in cases of generalized tuberculosis. The serous membranes lining the thorax and abdomen are easily torn out, and with them these tell-tale evidences of generalized infection. Enlarged lymphatic glands and abnormalities in appearance of the visceral organs will attract attention, by section of which the character of the disorder may be determined. It is differentiated from actinomycosis by the small yellowish points and the actinomyces grains of the latter disease, and from parasitic and other abscesses by the character of contents and the presence or absence of like lesions elsewhere, and if necessary, by aid of the microscope. In ordinary postmortem examinations in slaughtering establishments the inspector should have time to examine the entire carcass minutely in cases in which the gross lesions are confined to a single organ or gland; even then he cannot always discern whether generalized tuberculosis is in process of development from the localized form. Color, odor, texture, fatness or leanness give no hint to such extension of this disease. I have seen very fat carcasses which were actually studded with tubercles all over the external and internal surfaces, as well as being profusely interspersed throughout the muscular tissues. Of course, many cases are emaciated. Localized tuberculosis, whether it be in lymphatic or mammary gland, in lung or liver, does not apparently modify the physical appearance of the carcass.

There seems to be a great diversity of opinion as to the wholesomeness or unwholesomeness of the flesh of tuberculous cattle, even in Europe, where several international congresses have debated the subject at great length. The consensus of opinion seems to be that in all cases of generalized tuberculosis the carcass should be condemned, and when localized the flesh may be safely used for food.

The disease known as "Texas fever," or "Southern fever," may be recognized in living animals which have been driven to the abattoirs for slaughter, if they are allowed to become quiet, for as soon as the excitement of the drive is past a sick animal assumes a characteristic position. The back is arched, the limbs are spread apart to enable it to stand steadily, the head is dropped low, the ears fall downward and forward, or the animal may lie down, when the head is carried around to the flank, as in parturient apoplexy. If a thermometer be employed, it is usually found that the rectal temperature is from 103° to 106° F. Should the animal void urine, the dark wine-color will be very noticeable, and when the sick animal is made to walk, after a period of rest, a staggering gait will attract attention. If it be docile, an examination of the visible mucous membranes may be made, but in range cattle the prudent inspector will dispense with the information to be so acquired, for animals sick with Texas fever are more excitable and vicious than healthy cattle. The presence of ticks on the escutcheon, thighs, flanks, and elsewhere confirm the diagnosis.

When an animal sick with Texas fever is slaughtered the examiner will find the spleen greatly enlarged, its capsule easily torn, and the substance of the gland quite black and very soft, sometimes partly liquid, so that considerable of the splenic mass will gravitate to either end of the capsule if suspended by the other end. The liver is much enlarged and changed from a brownish to a mahogany color, also somewhat mottled on cut surfaces due to being irregularly stained with coloring matter from the blood. The gall-bladder is distended with a very dark, tarry, viscid bile, in which is suspended a quantity of yellow flakes which will deposit upon standing. The urine in the bladder has a dark-red to port-wine color, and the kidneys will be found congested. Other visceral organs present no characteristic lesions. In some carcasses the tissues have a yellowish tinge and the fat a bright lemon-yellow shade. In other carcasses the color of the flesh is normal, but the cancellous structure of the bones is stained dark like the urine.

The foregoing presents the principal antemortem symptoms and postmortem lesions of the acute disease fully developed. In this type of cases an inspector would not be in doubt as to whether or not an animal is diseased, nor as to what disease it is; neither would he hesitate concerning its condemnation. In the same bunch of cattle in which this typical case is found there will probably be others in which this malady is just beginning to develop or is partly developed. The structural changes in the spleen and liver are not so marked, perhaps scarcely discernible. The disintegration of blood-corpuscles may not be sufficient to stain the urine highly; or the case may be of a very mild type. It will tax the judgment of an inspector to determine rightly whether or not the animal is infected; whether or not the disease is sufficiently developed to render the flesh unwholesome, this disease not being communicable to man.

Advanced pregnancy and the parturient state, though normal conditions, should reject the cow for slaughter. Sentiment renders the flesh of such unappetizing, as well as the flesh of the unborn and recently born calf. Local regulations usually require the calf to be four to six weeks old or to weigh at least seventy-five pounds when dressed.

Extreme emaciation from any cause so modifies the tissues that the carcass does not become firm and dry in the refrigerator, like normal flesh, and accordingly is very deficient in nutritive quality, and should be rejected.

Leucocythæmia, or leukæmia, is occasionally found on the slaughter-beds. Enlargements of the lymphatic glands and spleen are the abnormalities which attract the attention of the inspector. In the several cases coming under my notice the animals were in thin flesh, presenting the appearance of general unthriftiness. The spleen was many times the normal size, and lymphatic glands in all parts of the body were from two to ten times the usual diameters; cross-sections presented normal appearance.

Non-specific inflammation of every viscus is occasionally found, and the disposition of the carcass must be determined by the stage of development and extent and character of perverted functional activity. It is conceded that high bodily temperature, long continued, impairs the quality and character of flesh, rendering it unappetizing, noisome, and suspiciously unwholesome.

Acute inflammation, as well as chronic structural changes of the kidneys, are quite apt to escape notice, owing to these organs being embedded in considerable fat. Any considerable interference with the renal function soon leaves the tissues charged with waste-products, which prevent the usual firming of the flesh, it remaining soft and sticky or clammy to the touch, and gives out a loathsome, urinous odor. Such flesh should be condemned.

Cold abscesses may be found in all parts of the animal carcass, but are most frequently found attached to a thoracic or abdominal viscus. There is very rarely more than one in any individual, and they vary greatly in size. They are most frequently found in young, highly-developed, and rapidly-fattened cattle which present every appearance of perfect healthfulness. They consist of a very dense limiting membrane inclosing a whitish, odorless purulent fluid which is rather gruesome to look upon. There is no morbid disturbance in the structures contiguous to these abscesses, and they can be enucleated, leaving the carcass wholesome food.

An occasional case of pyæmia, or multiple abscess, throughout the body is met with. Investigation usually reveals the source of infection in a suppurating wound or purulent inflammation of the uterus or serous membrane; these and cases of septic infection from a retained fœtus or placenta, or from a gangrenous organ or wound, will call for condemnation. The carcass in such cases gives out an offensive odor and does not dry and harden when placed in the cooler.

Genuine jaundice is seldom seen, and when found indicates condensation. A pseudo-jaundice is very abundant and is due to the peculiar coloring of fatty tissues. I have noticed that the fat of animals which are in a thriving and improving condition is yellowish white, and the fat of those in a retrograding condition is more highly colored, even acquiring a dark-orange yellow color, giving the carcass a jaundiced appearance.

Some Southern cattle are infected with flukes. These parasites may be sufficiently numerous to channel the liver in all portions and stimulate new growth of tissue-elements sufficiently to double or quadruple its normal size, yet the appearance of the carcass is normal and appetizing. The liver alone is rendered objectionable.

The cysticercus bovis is very rarely found in cattle coming from the region west of the Mississippi River. The cysts are usually most numerous in the muscles of the cheek; they are about the size of a navy bean, and consist ot a cyst wall inclosing a small tapeworm head and a quantity of limpid transparent fluid. The presence of this parasite (one source of tapeworm in man) indicates condemnation.

Sheep which come to the Western markets are less subject to disease than cattle and hogs. The most frequent cause for condemnation is emaciation. Some of the sheep from Mexico, New Mexico, and Colorado are infested with tapeworms, which are so numerous in the small intestines and bile-ducts that the nutritive functions are greatly interfered with. There is no fat and little muscle on the carcass, and that little is so devoid of the normal constituents that it remains soft and flabby under the same conditions in which the carcasses of healthy, well-nourished animals become dry and firm.

Jaundice is quite common, and is dependent upon pathological derangement of the liver, usually inflammation of that organ, but occasionally atrophy or sclerosis of the hepatic tissues is the cause. Some cases of jaundice found in sheep shipped from Western ranges are probably cases of ictero-hæmaturia; the spleen is large, the liver black and friable, bladder full of high-colored urine, the skin and other tissues stained intensely yellow.

A disease somewhat resembling tuberculosis is found in sheep grazed on the plains of Colorado and Utah. It is characterized by the development of caseous masses in the lungs and thoracic glands, the glandular masses often becoming two to three inches in diameter, and even greater. I do not remember to have seen the extension of this disease to any tissue or organ outside the thoracic cavity. The disease is essentially chronic and apparently does not interfere with the thriftiness of the animal until large areas of the lungs are invaded and destroyed. When the health of the animal has been impaired by this disease it would seem self-evident that the carcass should not be used as food. In all cases the organs invaded should be destroyed.

Another disease somewhat resembling tuberculosis is found in the walls of the intestines and in the mesenteric glands. It consists of nodules of various sizes, made up of adventitious tissue inclosing caseous pus, and sometimes there is found in addition to it small round-worms. This is known as the nodular disease, and its only apparent effect is the rendering of the intestines valueless as sausage casings. In these cases, as well as in all wounds, abscesses, septic conditions, advanced pregnancy, etc., the same rules for condemnation apply as in cattle.

Many sheep are the bearers of the cystic tænia marginata, which are mostly found attached to the folds of the peritoneum; as they are harmless to man the carcass is wholesome food, but butchers should be required to remove all cysts and put them into the furnace or retort (in order that they may not be thrown to dogs, in whose intestines they become mature tapeworms).

A few cases of scab have come under my notice, in which inflammatory processes extended beneath the skin. The animals were anæmic and apparently subjects of septic poison. These cases were condemned.

Swine are subject to bruise and fracture during transportation, also to many diseases identical with those of cattle, and the same principles apply in determining the wholesomeness of the flesh for food. There are some special diseases of swine, of which hog-cholera and swine-plague are the most important. These two diseases are frequently associated in the same animal, and bacteriological culture-tests are often necessary to determine which it is, if both diseases are not present.

It is the common custom of stock-owners to ship their herds to market when contagious diseases develop in them, regardless of their fitness or fatness, and sell them for what they will bring, in order to avoid a greater financial loss. More especially is this the case when the animals are swine affected with cholera. In the stage of invasion, or in mild cases, none of the physical signs are sufficiently marked to indicate the diseased hog when driven into the slaughter-pen, but in the more advanced stages of ordinary virulence the sick hog lags behind, has a staggering gait, may cough violently, and is so exhausted by a short drive that a spasmodic action of the diaphragm (commonly called thumps) is present in many cases. When allowed to stop the snout is dropped to the ground, the back arched, the abdomen tucked up, and vomiting, purging or both occur if the animal has access to water, which follows its endeavor to quench an insatiable thirst. Red discolorations of the skin are usually present in various parts, the ears are frequently swollen to two or three times the normal size, and thickness and occasionally necroses of the skin and subcutaneous tissues occur upon the ears and other parts which have been bruised. When slaughtered and the hair and cuticular layer of the skin are removed in the usual process of preparing the carcass for food, the hemorrhagic discolorations of the skin which are present in nearly all cases of cholera will attract the inspector's attention. This discoloration varies from a bright-red color in recent cases to a dark-gray pigmentation in convalescing cases. They may vary in size from small lenticular spots on the legs, jowl, and neck to blotches several inches in diameter situated on any part of the body.

Strokes of the whip or other light contusions of the skin will produce light-red marks in healthy hogs, but in cases of cholera the color is dark-red and extends a considerable distance from the injury.

Hemorrhages also occur into the subcutaneous fat from very slight contusions and show as dark spots under the skin. In cases of several days' standing these hemorrhagic areas often necrose, and an incision through the skin reveals a quantity of dirty, brown, putrid fluid. The overlying skin will slough if the animal lives long enough. The lymphatic glands in all parts of the body present hemorrhagic lesions, which vary from redness of the periphera to a dark, bloody discoloration of the entire glandular mass. Extravasations of blood beneath the serous membranes are often quite extensive, especially in the lungs, mesenteric folds, and leaf-lard. In mild cases the kidneys are studded with minute points of coagulated blood, and in violent cases the pelves and capsules may contain extensive clots of blood. The characteristic exudation nodes (buttons of Welch) and ulcerations of the intestinal mucous membrane are rarely difficult to find, especially in the region of the ileo-cæcal valve, and may be confidently looked for to confirm a doubtful diagnosis. The hemorrhagic lesions of the skin, lymphatic glands, and serous membranes are usually sufficiently marked to render a diagnosis certain, but these lesions are sometimes very slight, and an examination of the intestinal tract may be necessary to determine whether an incipient pneumonia or false membrane present in a given animal is due to cholera and swine-plague or other causes. The hemorrhagic lesions are sometimes not conspicuous, and may be readily overlooked when hogs are being slaughtered at the rate of 200 to 500 an hour. Swine-plague is usually manifested by a congested condition of the skin covering a large area, either one-half or two-thirds of the entire carcass, giving it the appearance of a deep-red blush. The internal lesions are most pronounced in the lungs, being a form of pneumonia in which yellowish points are discerned; these points are necrotic spots or centres. The serous surface of the lungs, as well as all other serous surfaces, may be covered with a fibrinous exudate, either in a thin layer or in many layers, and when the abdominal viscera is the region more generally involved, all of these viscera are agglutinated together. This condition ought readily to be discriminated from simple peritonitis, pleuritis, or pericarditis by involvement of the serous surfaces in other parts of the body and by the characteristic appearance of the lungs and skin. A diagnosis of hog-cholera or swine-plague should always mean condemnation of carcass and viscera.

Swine infested with cysticercus cellulosæ are found occasionally. In the few cases I have seen cysticerci were present in great number, pervading all the voluntary muscular structures and the heart. When found elsewhere the cysts were imperfectly developed. They appear as little sacs of water about one-fourth of an inch in diameter, lying upon and wedged between the muscular fibres. Each sac contains a white mass about the size of a millet-seed (a tapeworm-head) which projects from the cyst-wall. Flesh containing these cysts is commonly denominated "measly" pork, and is the source of tapeworm (tænia solium) in man. Of course, the flesh would be rendered harmless if thoroughly cooked, but would remain disgusting, and should be condemned.

The report of the Department of Agriculture states that 3.05 per cent. of all hogs examined microscopically by the department during the fiscal year ending June 30, 1893, were infested with trichinæ, and as the number examined exceeded 1,500,000, it is evident that this parasite is widespread and very prevalent. Trichinæ produce no gross lesions in the infested animal and are detected only by the aid of the microscope. They are found almost exclusively in the muscular structures, and are most numerous in the tongue, diaphragm, and psoas muscles, but are confined to no section of the carcass. They are readily detected when magnified thirty to sixty diameters, and specimens of muscle, either fresh or cured, are easily prepared for examination, either by mincing or cutting into small pieces and spreading thin enough to permit light to pass through. The trichinæ are found coiled like spiral springs and are inclosed in sacs of transparent fluid, usually one in a cyst, but sometimes two or three, or even five or six. The cyst and contents, including the worm, are subject to both fatty and calcareous degeneration ; in the latter form of degeneration the trichina is often black and fragile, being frequently broken into fragments in the preparation. Trichinized flesh does not differ from the non-infested flesh in appearance, and is harmful as food only when eaten uncooked. The communities and nations which eat their pork raw naturally require the inspection and condemnation of trichinized pork.

The cystic form of the echinococcus veterinorum is very common in swine, and the hydatids are found almost exclusively in the liver. While the authors have reports of finding this cystic parasite in all parts of the body of both man and animals, medical and veterinary records do not show such a widespread diffusion in this section of the country. The cysts vary in size from one-quarter inch to two or three inches in diameter and consist of a translucent double wall inclosing its full capacity of transparent liquid. The inner wall (mother membrane) is easily separated from the outer wall, and if divided it persistently rolls upon itself when effort is made to spread it flat upon a surface. The inner surface of this wall or membrane usually bears many minute whitish bodies, only observable upon close examination. These bodies are made up of from ten to twenty tapeworm-heads, which are plainly visible upon fifty to one hundred diameters, magnification. The cysts and contents are modified by degenerate processes and may be converted into abscesses. They are found on the surface or embedded in the substance of the liver and vary in number from one to many. Infested organs should be rendered unusable as food for man or beast.

This presentation in short review of the gross diagnostic lesions of disease and conditions of food-animals, and comments as to the disposition of the flesh, is all too brief, but may serve to open the discussion. Diseases and conditions which have not come under the writer's personal observation have been purposely omitted.

